# Efficient class of estimators for finite population mean using auxiliary attribute in stratified random sampling

**DOI:** 10.1038/s41598-023-34603-z

**Published:** 2023-06-24

**Authors:** Housila P. Singh, Anurag Gupta, Rajesh Tailor

**Affiliations:** 1 School of Studies in Statistics, Vikram University, Ujjain, M.P. 456010 India; 2 Indian Agricultural Statistics Research Institute, ICAR, New Delhi, 110012 India

**Keywords:** Applied mathematics, Statistics

## Abstract

The aim of this paper is to develop more effective methods for estimating population means in sample surveys using auxiliary attributes. To achieve this goal, we introduce a modified version of the estimators proposed by Koyuncu (2013b) and Shahzad et al. (2019), as well as a new class of estimators. We derive expressions for the bias and mean squared error of these new estimators up to the first degree of approximation. Our results show that the suggested classes of estimators perform better than other existing methods, with the lowest mean squared error under optimal conditions. We also conduct an empirical investigation to support our findings.

## Introduction

The use of auxiliary attribute is a well-known method for improving the efficiency of an estimator in estimating population parameters. Auxiliary attributes (say $\phi$), which are highly correlated with the study variable (*y*), are commonly encountered in practice. Examples include a person's height (*y*), the amount of milk produced by a cow (*y*), and the yield of a particular variety of wheat (*y*), which may respectively depend on factors such as gender, breed of the cow, and type of wheat. For more examples, see Kendale and Stuart^1^, Shabbir and Gupta^2^, and Sharma and Singh^3^, among others. The estimation of the population mean of the study variable (*y*) using an auxiliary attribute $\left( \phi \right)$ under simple random sampling without replacement has been extensively studied. See, for instance, Naik and Gupta^4^, Jhajj et al.^5^, Solanki and Singh^6^, Singh et al.^7^, Gupta and Tailor^8^, and other relevant literature.

The simple random sampling scheme is commonly used when the population units are homogeneous. However, in many practical situations, the population units are heterogeneous, and to obtain a better estimate of the population parameters, we use stratified random sampling. Therefore, our objective is to estimate the population mean of the study variable (*y*) using information on an auxiliary attribute $\left( \phi \right)$ under stratified random sampling. Various authors, including Sharma and Singh^3^, Koyuncu^9,10^, Shahzad et al.^11^, Zaman^12,13^, Hussain et al.^14,15^, Zaman et al.^16,17^, and Ahmad et al.^18,19^, have discussed the problem of estimating the population mean of the study variable using an auxiliary attribute under stratified random sampling.

Shahzad et al.^20,21^ used a calibration approach in stratified random sampling. They also proposed an estimator to estimate the coefficient of variation using calibrated estimators in stratified random sampling Shahzad et al.^22^.

### Notations

Consider a population size *N* unit, is divided into *L* strata units *h*th stratum containing *N*_*h*_ units, where *h* = 1, 2,…, *L* such that ${\sum }_{h=1}^{L}{N}_{h}=N$. A simple random sample of size *n*_*h*_ is drawn without replacement the *h*th stratum such that ${\sum }_{h=1}^{L}{n}_{h}=n$. Let $\left({y}_{hi},{\phi }_{hi}\right)$ be observed value of study variable *y* and the auxiliary attribute $\phi$ on the *i*th unit of the *h*th stratum, respectively, where $i=\mathrm{1,2},...,{N}_{h}$ and $h=\mathrm{1,2},...,L$.

Further, let ${\overline{y}}_{h}={\sum }_{h=1}^{{n}_{h}}\frac{{y}_{hi}}{{n}_{h}}$ and ${\overline{y}}_{st}={\sum }_{h=1}^{L}{W}_{h}{\overline{y}}_{h}$ be unbiased estimators of population means ${\overline{Y}}_{h}={\sum }_{i=1}^{{N}_{h}}\frac{{y}_{hi}}{{N}_{h}}$ and $\overline{Y}={\sum }_{h=1}^{L}{W}_{h}{\overline{Y}}_{h}$, where ${W}_{h}=\frac{{N}_{h}}{N}$ is the stratum weight.

We also assume that

$$\phi_{hi} = \left\{ {\begin{array}{ll} {1, \;\;\; i{^\text{th}}\,{\text{unit}}\,{\text{of}}\,{\text{the}}\,h{^\text{th}}\,{\text{stratum}}\,{\text{possesses}}\,{\text{the}}\,{\text{attribute}}\,\phi ,} \\ {0, \;\;\; {\text{otherwise}}{.}\,\,\,\,\,\,\,\,\,\,\,\,\,\,\,\,\,\,\,\,\,\,\,\,\,\,\,\,\,\,\,\,\,\,\,\,\,\,\,\,\,\,\,\,\,\,\,\,\,\,\,\,\,\,\,\,\,\,\,\,\,\,\,\,\,\,\,\,\,\,\,\,\,\,\,\,\,\,\,\,} \\ \end{array} } \right.$$

Let ${s}_{yh}^{2}={\sum }_{i=1}^{{n}_{h}}\frac{{\left({y}_{hi}-{\overline{y}}_{h}\right)}^{2}}{\left({n}_{h}-1\right)}$ and ${s}_{\phi h}^{2}={\sum }_{i=1}^{{n}_{h}}\frac{{\left({\phi }_{hi}-{p}_{h}\right)}^{2}}{\left({n}_{h}-1\right)}$ be the *h*th sample variances and ${S}_{yh}^{2}={\sum }_{i=1}^{{N}_{h}}\frac{{\left({y}_{hi}-{\overline{Y}}_{h}\right)}^{2}}{\left({N}_{h}-1\right)}$ and ${S}_{\phi h}^{2}={\sum }_{i=1}^{{N}_{h}}\frac{{\left({\phi }_{hi}-{P}_{h}\right)}^{2}}{\left({N}_{h}-1\right)}$ be the *h*th population variances of the study variable *y* and the auxiliary attribute $\phi$, respectively.

Further, ${s}_{y\phi h}={\sum }_{i=1}^{{n}_{h}}\frac{\left({y}_{hi}-{\overline{y}}_{h}\right)\left({\phi }_{hi}-{p}_{h}\right)}{\left({n}_{h}-1\right)}$ and ${\widehat{\rho }}_{y\phi h}=\frac{{s}_{y\phi h}}{{s}_{yh}{s}_{\phi h}}$ are the *h*th sample covariance and point bi-serial correlation and ${S}_{y\phi h}={\sum }_{i=1}^{{N}_{h}}\frac{\left({y}_{hi}-{\overline{Y}}_{h}\right)\left({\phi }_{hi}-{P}_{h}\right)}{\left({N}_{h}-1\right)}$ and ${\widehat{\rho }}_{y\phi h}=\frac{{S}_{y\phi h}}{{S}_{yh}{S}_{\phi h}}$ are population covariance and point bi-serial correlation between the study variable *y* and the auxiliary attribute $\phi$, respectively.

To derive the bias and mean squared error (*MSE*) of the estimators, we write

$$\overline{y}_{st} = \overline{Y}\left( {1 + e_{o} } \right),\;p_{st} = P\left( {1 + e_{1} } \right)$$

such that $E\left( {e_{o} } \right) = E\left( {e_{1} } \right) = 0$ and

$$E\left({e}_{0}^{2}\right)=\frac{1}{{\overline{Y}}^{2}}\sum \limits_{h=1}^{L}{W}_{h}^{2}{\gamma }_{h}{S}_{yh}^{2}={A}_{y},$$

$$E\left({e}_{1}^{2}\right)=\frac{1}{{P}^{2}}\sum \limits_{h=1}^{L}{W}_{h}^{2}{\gamma }_{h}{S}_{\phi h}^{2}={A}_{\phi },$$

$$E\left({e}_{0}{e}_{1}\right)=\frac{1}{\overline{Y}P}\sum \limits_{h=1}^{L}{W}_{h}^{2}{\gamma }_{h}{S}_{y\phi h}={A}_{y\phi },$$

where ${p}_{st}={\sum }_{h=1}^{L}{W}_{h}{p}_{h}$ such that $E\left({p}_{st}\right)=P={\sum }_{h=1}^{L}{W}_{h}{P}_{h}$ and ${\gamma }_{h}=\frac{{N}_{h}-{n}_{h}}{{n}_{h}{N}_{h}}$.

### Reviewing some existing estimators

The conventional unbiased estimators for population mean $\overline{Y}$ of the study variable *y* under stratified random sampling is given by

1 $${t}_{0}={\overline{y}}_{st}=\sum \limits_{h=1}^{L}{W}_{h}{\overline{y}}_{h}$$

The variance/*MSE* of the estimator $t_{0}$ is given by

2 $$MSE\left({t}_{0}\right)=\sum \limits_{h=1}^{L}{W}_{h}^{2}{\gamma }_{h}{S}_{yh}^{2}={\overline{Y}}^{2}{A}_{y} .$$

The ratio estimator for population mean $\overline{Y}$ using auxiliary attribute $\phi$ in stratified random sampling due to Naik and Gupta^4^ is given by

3 $${t}_{1}={\overline{y}}_{st}\left(\frac{P}{{p}_{st}}\right).$$

The *MSE* of the estimator $t_{1}$ up to the first degree of approximation (*fda*), is given by

4 $$MSE\left({t}_{1}\right)={\overline{Y}}^{2}\left[{A}_{y}+{A}_{\phi }\left(1-2C\right)\right],$$

where $$C=\frac{{A}_{y\phi }}{{A}_{\phi }}$$.

The stratified version of ordinary product estimator for population mean $\overline{Y}$ is defined by

5 $${t}_{2}={\overline{y}}_{st}\left(\frac{{p}_{st}}{P}\right).$$

The *MSE* of $t_{2}$ to the *fda*, is given by

6 $$MSE\left({t}_{2}\right)={\overline{Y}}^{2}\left[{A}_{y}+{A}_{\phi }\left(1+2C\right)\right].$$

The usual regression estimator for $\overline{Y}$ is given by

7 $${t}_{3}={\overline{y}}_{st}+{b}_{st}\left(P-{p}_{st}\right),$$

where $b_{st}$ is the sample regression coefficient of *y* on $\phi$.

To the *fda* the *MSE* of $t_{3}$ is given by

8 $$MSE\left({t}_{3}\right)={\overline{Y}}^{2}{A}_{y}\left(1-{\rho }_{y\phi }^{2}\right),$$

where $${\rho }_{y\phi }=\frac{{A}_{y\phi }}{\sqrt{{A}_{y}{A}_{\phi }}}$$.

Koyuncu^9^ suggested a class of estimators for $\overline{Y}$ is given by

9 $${t}_{4}=\left[{w}_{1}{\overline{y}}_{st}+{w}_{2}\left(P-{p}_{st}\right)\right]\left\{\frac{{a}_{st}P+{b}_{st}}{{a}_{st}{p}_{st}+{b}_{st}}\right\},$$

where $\left({w}_{1},{w}_{2}\right)$ are suitable constants, ${a}_{st}\left(\ne 0\right)$ and ${b}_{st}$ are either real numbers or the functions of the known parameters for the *h*th stratum of the auxiliary attribute $\phi$, such as standard deviation ${S}_{\phi \left(st\right)}={\sum }_{h=1}^{L}{W}_{h}{\sigma }_{\phi h}$, coefficient of variation ${C}_{\phi \left(st\right)}={\sum }_{h=1}^{L}{W}_{h}{C}_{\phi h}$ with ${C}_{\phi h}=\frac{{S}_{\phi h}}{{P}_{h}}$,skewness ${\beta }_{1\left(\phi \right)st}={\sum }_{h=1}^{L}{W}_{h}{\beta }_{1h}\left(\phi \right)$ and kurtosis ${\beta }_{2\left(\phi \right)st}={\sum }_{h=1}^{L}{W}_{h}{\beta }_{2h}\left(\phi \right)$ and correlation coefficient ${\rho }_{\left(y\phi \right)st}={\sum }_{h=1}^{L}{W}_{h}{\rho }_{y\phi {h}}$ where ${\beta }_{1h\left(\phi \right)}=\frac{{\mu }_{3h}^{2}\left(\phi \right)}{{\mu }_{2h}^{3}\left(\phi \right)}$, ${\beta }_{2h\left(\phi \right)}=\frac{{\mu }_{4h}\left(\phi \right)}{{\mu }_{2h}^{2}\left(\phi \right)}$, ${\sigma }_{\phi h}^{2}={\mu }_{2h}\left(\phi \right)=\frac{1}{{N}_{h}}{\sum }_{i=1}^{{N}_{h}}{\left({\phi }_{hi}-{P}_{h}\right)}^{2}$, ${\mu }_{3h}\left(\phi \right)=\frac{1}{{N}_{h}}{\sum }_{i=1}^{{N}_{h}}{\left({\phi }_{hi}-{P}_{h}\right)}^{3}$ and ${\mu }_{4h}\left(\phi \right)=\frac{1}{{N}_{h}}{\sum }_{i=1}^{{N}_{h}}{\left({\phi }_{hi}-{P}_{h}\right)}^{4}$.

The *MSE* of the estimator *t*_4_ to the *fda* is given by

10 $$MSE\left({t}_{4}\right)={\overline{Y}}^{2}\left[1+{w}_{1}^{2}{B}_{1}+{w}_{2}^{2}{B}_{2}+2{w}_{1}{w}_{2}{B}_{3}-2{w}_{1}{B}_{4}-2{w}_{2}{B}_{5}\right],$$

where $${B}_{1}=\left[1+{A}_{y}+{\upsilon }_{st}{A}_{\phi }\left(3{\upsilon }_{st}-4C\right)\right]$$, $${B}_{2}=\frac{{A}_{\phi }}{{R}^{2}},$$ $${B}_{3}=\left(\frac{{A}_{\phi }}{R}\right)\left(2{\upsilon }_{st}-C\right),$$ $${B}_{4}=\left[1+{\upsilon }_{st}{A}_{\phi }\left({\upsilon }_{st}-C\right)\right],$$ $${B}_{5}=\left(\frac{{A}_{\phi }}{R}\right){\upsilon }_{st}$$,$$R=\frac{\overline{Y}}{P}$$ and $${\upsilon }_{st}=\frac{{a}_{st}P}{\left({a}_{st}P+{b}_{st}\right)}$$.

The *MSE t*_4_ at (10) is minimized for

11 $$\left.\begin{array}{c}{w}_{1}=\frac{\left({B}_{2}{B}_{4}-{B}_{3}{B}_{5}\right)}{\left({B}_{1}{B}_{2}-{B}_{3}^{2}\right)}\\ {w}_{2}=\frac{\left({B}_{1}{B}_{5}-{B}_{3}{B}_{4}\right)}{\left({B}_{1}{B}_{2}-{B}_{3}^{2}\right)}\end{array}\right\}.$$

Therefore, the resulting minimum *MSE* of *t*_*4*_ is given by

12 $$\begin{aligned} MSE_{\min } \left( {t_{4} } \right) & = \overline{Y}^{2} \left[ {1 - \frac{{\left( {B_{2} B_{4}^{2} - 2B_{3} B_{4} B_{5} + B_{1} B_{5}^{2} } \right)}}{{\left( {B_{1} B_{2} - B_{3}^{2} } \right)}}} \right] \hfill \\ & = \overline{Y}^{2} \left[ {1 - \frac{{\left( {A_{\phi } - \upsilon_{st}^{2} A_{\phi } A_{y\phi }^{2} - \upsilon_{st}^{2} A_{\phi }^{2} + \upsilon_{st}^{2} A_{\phi }^{2} A_{y}^{{}} } \right)}}{{\left( {A_{\phi } + A_{y} A_{\phi } - \upsilon_{st}^{2} A_{\phi }^{2} - A_{y\phi }^{2} } \right)}}} \right]. \hfill \\ \end{aligned}$$

Using information on auxiliary attribute $\phi$, Sharma and Singh^3^ proposed the following exponential type estimators for $\overline{Y}$ as

13 $${t}_{1e}={\overline{y}}_{st}\mathit{exp}\left(\frac{P-{p}_{st}}{P+{p}_{st}}\right),\; (\mathrm{ratio \; type \; exponential \; estimator})$$

14 $${t}_{2e}={\overline{y}}_{st}\mathit{exp}\left(\frac{{p}_{st}-P}{{p}_{st}+P}\right), \; (\text{product-type exponential estimator})$$

15 $${t}_{\alpha e}={\overline{y}}_{st}\mathit{exp}\left\{\frac{\alpha \left(P-{p}_{st}\right)}{\left(P+{p}_{st}\right)}\right\},$$

where $\alpha$ being a suitable chosen constant.

To the *fda* the *MSE*s of the estimators $t_{1e,} t_{2e}$ and $t_{\alpha e,}$ are respectively given by

16 $$MSE\left( {t_{1e} } \right) = \overline{Y}^{2} \left[ {A_{y} + \frac{{A_{\phi } }}{4}\left( {1 - 4C} \right)} \right],$$

17 $$MSE\left( {t_{2e} } \right) = \overline{Y}^{2} \left[ {A_{y} + \frac{{A_{\phi } }}{4}\left( {1 + 4C} \right)} \right],$$

18 $$MSE\left( {t_{\alpha e} } \right) = \overline{Y}^{2} \left[ {A_{y} + \frac{{\alpha A_{\phi } }}{4}\left( {\alpha - 4C} \right)} \right].$$

The $MSE\left({t}_{\alpha e}\right)$ is minimum when

19 $$\alpha =2C.$$

This yields the minimum *MSE* of ${t}_{\alpha e}$ is given by

20 $$MSE_{\min } \left( {t_{\alpha e} } \right) = \overline{Y}^{2} A_{y} \left( {1 - \rho_{y\phi }^{2} } \right).$$

Sharma and Singh^3^ proposed the following class of estimators for population mean $\overline{Y}$ as

21 $${t}_{5}=\left[{w}_{1}{\overline{y}}_{st}+{w}_{2}\left(P-{p}_{st}\right)\right]\mathit{exp}\left\{\frac{{a}_{st}\left(P-{p}_{st}\right)}{{a}_{st}\left(P+{p}_{st}\right)+2{b}_{st}}\right\},$$

where $\left({a}_{st},{b}_{st}\right)$ are same as defined for the class of estimators ${t}_{4}$ at (9) and $\left({w}_{1},{w}_{2}\right)$ are suitable chosen constants to be determined such that *MSE* of ${t}_{5}$ is minimum.

If we set ${a}_{st}=1$ and ${b}_{st}=NP$ in (21), then the class of estimators ${t}_{5}$ reduces to the Shahzad et al.^11^ class of estimators for $\overline{Y}$ as

22 $${t}_{6}=\left[{w}_{1}{\overline{y}}_{st}+{w}_{2}\left(P-{p}_{st}\right)\right]\mathit{exp}\left\{\frac{\left(P-{p}_{st}\right)}{P+{p}_{st}+2NP}\right\}.$$

We note that the expressions of bias and *MSE* of the class of estimators ${t}_{5}$ derived by Sharma and Singh^3^ [Eqs. (4.6) and (4.7), p. 1789] are not correct. The correct expressions of bias and *MSE* of the estimator ${t}_{5}$ to the *fda* are respectively given by

23 $$B\left({t}_{5}\right)=\overline{Y}\left({w}_{1}{A}_{4}+{w}_{2}{A}_{5}-1\right),$$

and

24 $$MSE\left({t}_{5}\right)={\overline{Y}}^{2}\left[1+{w}_{1}^{2}{A}_{1}+{w}_{2}^{2}{A}_{2}+2{w}_{1}{w}_{2}{A}_{3}-2{w}_{1}{A}_{4}-2{w}_{2}{A}_{5}\right],$$

where $${A}_{1}=\left[1+{A}_{y}+{\upsilon }_{st}{A}_{\phi }\left({\upsilon }_{st}-2C\right)\right]$$, $${A}_{2}=\frac{{A}_{\phi }}{{R}^{2}}$$, $${A}_{3}=\left(\frac{1}{R}\right){A}_{\phi }\left({\upsilon }_{st}-C\right)$$, $${A}_{4}=\left[1+\frac{{\upsilon }_{st}{A}_{\phi }}{8}\left(3{\upsilon }_{st}-4C\right)\right]$$, $${A}_{5}=\frac{{\upsilon }_{st}{A}_{\phi }}{2R}$$.

The *MSE t*_5_ at (24) is minimized for

25 $$\left.\begin{array}{c}{w}_{1}=\frac{{A}_{2}{A}_{4}-{A}_{3}{A}_{5}}{{A}_{1}{A}_{2}-{A}_{3}^{2}}\\ {w}_{2}=\frac{{A}_{1}{A}_{5}-{A}_{3}{A}_{4}}{{A}_{1}{A}_{2}-{A}_{3}^{2}}\end{array}\right\}.$$

Therefore, the minimum *MSE* of *t*_*5*_ is given by

26 $$MSE_{\min } \left( {t_{5} } \right) = \overline{Y}^{2} \left[ {1 - \frac{{A_{2} A_{4}^{2} - 2A_{3} A_{4} A_{5} + A_{1} A_{5}^{2} }}{{A_{1} A_{2} - A_{3}^{2} }}} \right]$$

Putting ${a}_{st}=1$ and ${b}_{st}=NP$ in (24), we get the *MSE* of $t_{6}$ to the *fda* is given by

27 $$MSE\left({t}_{6}\right)={\overline{Y}}^{2}\left[1+{w}_{1}^{2}{A}_{1\left(1\right)}+{w}_{2}^{2}{A}_{2\left(1\right)}+2{w}_{1}{w}_{2}{A}_{3\left(1\right)}-2{w}_{1}{A}_{4\left(1\right)}-2{w}_{2}{A}_{5\left(1\right)}\right],$$

where $${A}_{1\left(1\right)}=\left[1+{A}_{y}+{\upsilon }_{st\left(1\right)}{A}_{\phi }\left({\upsilon }_{st\left(1\right)}-2C\right)\right]$$, $${A}_{2\left(1\right)}=\frac{{A}_{\phi }}{{R}^{2}}$$, $${A}_{3\left(1\right)}=\left(\frac{1}{R}\right){A}_{\phi }\left({\upsilon }_{st\left(1\right)}-C\right)$$, $${A}_{4\left(1\right)}=\left[1+\frac{{\upsilon }_{st\left(1\right)}{A}_{\phi }}{8}\left(3{\upsilon }_{st\left(1\right)}-4C\right)\right]$$, $${A}_{5\left(1\right)}=\frac{{\upsilon }_{st\left(1\right)}{A}_{\phi }}{2R}$$,$${\upsilon }_{st\left(1\right)}=\frac{1}{\left(N+1\right)}$$.

The *MSE t*_6_ at (27) is minimum when

28 $$\left.\begin{array}{c}{w}_{1}=\frac{{A}_{2\left(1\right)}{A}_{4\left(1\right)}-{A}_{3\left(1\right)}{A}_{5\left(1\right)}}{{A}_{1\left(1\right)}{A}_{2\left(1\right)}-{A}_{3\left(1\right)}^{2}}\\ {w}_{2}=\frac{{A}_{1\left(1\right)}{A}_{5\left(1\right)}-{A}_{3\left(1\right)}{A}_{4\left(1\right)}}{{A}_{1\left(1\right)}{A}_{2\left(1\right)}-{A}_{3\left(1\right)}^{2}}\end{array}\right\}.$$

Therefore, the minimum *MSE* of *t*_*6*_ is given by

29 $$MSE_{\min } \left( {t_{6} } \right) = \overline{Y}^{2} \left[ {1 - \frac{{A_{2\left( 1 \right)} A_{4\left( 1 \right)}^{2} - 2A_{3\left( 1 \right)} A_{4\left( 1 \right)} A_{5\left( 1 \right)} + A_{1\left( 1 \right)} A_{5\left( 1 \right)}^{2} }}{{A_{1\left( 1 \right)} A_{2\left( 1 \right)} - A_{3\left( 1 \right)}^{2} }}} \right].$$

Koyuncu^10^ and Shahzad et al.^11^ proposed the following class of estimators for $\overline{Y}$ as

30 $${t}_{7}=\left[{w}_{1}{\overline{y}}_{st}+{w}_{2}{\left(\frac{{p}_{st}}{P}\right)}^{\gamma }\right]\mathit{exp}\left\{\frac{{a}_{st}\left(P-{p}_{st}\right)}{{a}_{st}\left(P+{p}_{st}\right)+2{b}_{st}}\right\},$$

where $\left({w}_{1},{w}_{2},\gamma \right)$ are suitable chosen constants and $\left({a}_{st},{b}_{st}\right)$ are same as defined earlier.

To the *fda*, the *MSE* of *t*_7_ is given by

31 $$MSE\left({t}_{7}\right)={\overline{Y}}^{2}\left[1+{w}_{1}^{2}{C}_{1}+{w}_{2}^{2}{C}_{2}+2{w}_{1}{w}_{2}{C}_{3}-2{w}_{1}{C}_{4}-2{w}_{2}{C}_{5}\right],$$

where $${C}_{1}=\left[1+{A}_{y}+{\upsilon }_{st}{A}_{\phi }\left({\upsilon }_{st}-2C\right)\right]$$, $${C}_{2}=\frac{1}{{P}^{2}{R}^{2}}\left[1+{A}_{\phi }\left\{{\gamma }^{2}+{\upsilon }_{st}^{2}-2\gamma {\upsilon }_{st}+\gamma \left(\gamma -1\right)\right\}\right]$$, $${C}_{3}=\left(\frac{1}{PR}\right)\left[1+{A}_{\phi }\left\{\left({\upsilon }_{st}^{2}+\frac{\gamma \left(\gamma -1\right)}{2}-{\upsilon }_{st}\gamma \right)+\left(\gamma -{\upsilon }_{st}\right)C\right\}\right]$$, $${C}_{4}=\left[1+\frac{{\upsilon }_{st}{A}_{\phi }}{8}\left(3{\upsilon }_{st}-4C\right)\right]$$, $${C}_{5}=\frac{1}{PR}\left[1+\left\{\frac{\gamma \left(\gamma -1\right)}{2}-\frac{\gamma {\upsilon }_{st}}{2}+\frac{3}{8}{\upsilon }_{st}^{2}\right\}{A}_{\phi }\right]$$.

$MSE\left({t}_{7}\right)$ at (31) is minimized for

32 $$\left.\begin{array}{c}{w}_{1}=\frac{{C}_{2}{C}_{4}-{C}_{3}{C}_{5}}{{C}_{1}{C}_{2}-{C}_{3}^{2}}\\ {w}_{2}=\frac{{C}_{1}{C}_{5}-{C}_{3}{C}_{4}}{{C}_{1}{C}_{2}-{C}_{3}^{2}}\end{array}\right\}.$$

Therefore, the minimum *MSE* of *t*_*7*_ is given by

33 $$MSE_{\min } \left( {t_{7} } \right) = \overline{Y}^{2} \left[ {1 - \frac{{C_{2} C_{4}^{2} - 2C_{3} C_{4} C_{5} + C_{1} C_{5}^{2} }}{{C_{1} C_{2} - C_{3}^{2} }}} \right]$$

In this paper we have suggested a class of estimators for population mean $\overline{Y}$ of the study variable *y* using auxiliary attribute $\phi$. Expressions of bias and *MSE* of the proposed class of estimators are obtained up to terms of order 0 (n^−1^).

We have obtained the optimum condition under which the *MSE* of the proposed class of estimators is minimum. We have derived the conditions under which the suggested class of estimators is more efficient than the conventional estimator and the estimators due to Naik and Gupta^4^, Koyuncu^9^, Sharma and Singh^3^ and Shahzad et al.^11^. Numerical illustration is given in support of the proposed study.

## Suggested class of estimators

We note that the exponent part of (30) is obtained on using the transformation $\left({a}_{st}{p}_{st}+{b}_{st}\right)$ such that $E\left\{{a}_{st}{p}_{st}+{b}_{st}\right\}=\left({a}_{st}P+{b}_{st}\right)$, in the first bracket coefficient of ${w}_{2}$ is ${\left(\frac{{p}_{st}}{P}\right)}^{\gamma }$ which does not use the transformation $\left({a}_{st}{p}_{st}+{b}_{st}\right)$. Thus, authors are in opinion that coefficient of ${w}_{2}$ should be ${\left(\frac{{a}_{st}{p}_{st}+{b}_{st}}{{a}_{st}P+{b}_{st}}\right)}^{\gamma }$. Hence the modified suggested class of estimators for $\overline{Y}$ is given by

34 $$t_{7\left( m \right)} = \left[ {w_{1} \overline{y}_{st} + w_{2} \left( {\frac{{a_{st} p_{st} + b_{st} }}{{a_{st} P + b_{st} }}} \right)^{\gamma } } \right]\exp \left\{ {\frac{{a_{st} \left( {P - p_{st} } \right)}}{{a_{st} \left( {P + p_{st} } \right) + 2b_{st} }}} \right\}.$$

where $\left( {w_{1} ,w_{2} } \right)$ are suitably chosen constants to be determined such that *MSE* of $t_{7\left( m \right)}$ is minimum; and $\left( {a_{st} ,b_{st} ,\gamma } \right)$ are same as defined earlier.

To the *fda*, the bias and *MSE* of $t_{7\left( m \right)}$ are respectively given by

35 $$B\left( {t_{7\left( m \right)} } \right) = \overline{Y}\left( {w_{1} D_{4} + w_{2} D_{5} - 1} \right),$$

and

36 $$MSE\left( {t_{7\left( m \right)} } \right) = \overline{Y}^{2} \left[ {1 + w_{1}^{2} D_{1} + w_{2}^{2} D_{2} + 2w_{1} w_{2} D_{3} - 2w_{1} D_{4} - 2w_{2} D_{5} } \right],$$

where $$D_{1} = \left[ {1 + A_{y} + \upsilon_{st} A_{\phi } \left( {\upsilon_{st} - 2C} \right)} \right]$$, $$D_{2} = \,\frac{1}{{R^{2} P^{2} }}\left[ {1 + \upsilon_{st}^{2} \theta \left( {2\theta - 1} \right)A_{\phi } } \right]$$, $$D_{3} = \left( \frac{1}{RP} \right)\left[ {1 + \frac{{\upsilon_{st}^{{}} \left( {2\theta - 1} \right)}}{8}A_{\phi } \left( {2\theta + 4C - 3} \right)} \right]$$, $$D_{4} = \left[ {1 + \frac{{\upsilon_{st}^{{}} }}{8}A_{\phi } \left( {3\upsilon_{st}^{{}} - 4C} \right)} \right]$$, $$D_{5} = \frac{1}{RP}\left[ {1 + \frac{{\upsilon_{st}^{2} \theta \left( {\theta - 1} \right)}}{2}A_{\phi } } \right]$$, $$\theta = \frac{{\left( {2\gamma - 1} \right)}}{2}$$.

The $MSE\left( {t_{7\left( m \right)} } \right)$ at (36) is minimized for

37 $$\left. \begin{gathered} w_{1} = \frac{{\left( {D_{2} D_{4} - D_{3} D_{5} } \right)}}{{\left( {D_{1} D_{2} - D_{3}^{2} } \right)}} \hfill \\ w_{2} = \frac{{\left( {D_{1} D_{5} - D_{3} D_{4} } \right)}}{{\left( {D_{1} D_{2} - D_{3}^{2} } \right)}} \hfill \\ \end{gathered} \right\}\,.$$

Therefore, the minimum *MSE* of $t_{7\left( m \right)}$ is given by

38 $$MSE_{\min } \left( {t_{7\left( m \right)} } \right) = \overline{Y}^{2} \left[ {1 - \frac{{\left( {D_{2} D_{4}^{2} - 2D_{3} D_{4} D_{5} + D_{1} D_{5}^{2} } \right)}}{{\left( {D_{1} D_{2} - D_{3}^{2} } \right)}}} \right]$$

Now we can conclude this as a theorem given below.

### **Theorem 2.1**

*The* *MSE* *of* $t_{7\left( m \right)}$ *is greater than or equal to the minimum* *MSE* *of* $t_{7\left( m \right)}$.

$$MSE\left( {t_{7\left( m \right)} } \right) \ge MSE_{\min } \left( {t_{7\left( m \right)} } \right)$$

## An alternative class of estimators

We propose another class of estimators for population mean as $\overline{Y}$ as

39 $$t_{8} = \left[ {w_{1} \overline{y}_{st} + w_{2} \exp \left\{ {\frac{{\delta a_{st} \left( {P - p_{st} } \right)}}{{a_{st} \left( {P + p_{st} } \right) + 2b_{st} }}} \right\}} \right]\left( {\frac{{a_{st} P + b_{st} }}{{a_{st} p_{st} + b_{st} }}} \right)^{\eta } .$$

where $\left( {w_{1} ,w_{2} ,a_{st} ,b_{st} } \right)$ are same as defined earlier and $\left( {\delta ,\eta } \right)$ are constants which take real numbers like (− 1,0,1).

To the *fda*, the bias and *MSE* of $t_{8}$ are respectively given by

40 $$B\left( {t_{8} } \right) = \overline{Y}\left( {w_{1} E_{4} + w_{2} E_{5} - 1} \right),$$

and

41 $$MSE\left( {t_{8} } \right) = \overline{Y}^{2} \left[ {1 + w_{1}^{2} E_{1} + w_{2}^{2} E_{2} + 2w_{1} w_{2} E_{3} - 2w_{1} E_{4} - 2w_{2} E_{5} } \right],$$

where $$E_{1} = \left[ {1 + A_{y} - 4\eta \upsilon_{st} A_{y\phi } + \eta \left( {2\eta + 1} \right)\upsilon_{st}^{2} A_{\phi } } \right]$$, $$E_{2} = \,\frac{1}{{R^{2} P^{2} }}\left[ {1 + \theta \left( {2\theta + 1} \right)\upsilon_{st}^{2} A_{\phi } } \right]$$, $$E_{3} = \left( \frac{1}{RP} \right)\left[ {1 + \frac{{\left( {\eta + \theta } \right)\left( {\eta + \theta + 1} \right)}}{2}\upsilon_{st}^{2} A_{\phi } - \left( {\eta + \theta } \right)\upsilon_{st}^{{}} A_{y\phi } } \right]$$, $$E_{4} = \left[ {1 + \frac{{\eta \upsilon_{st}^{{}} }}{2}\left\{ {\frac{{\left( {\eta + 1} \right)}}{2}\upsilon_{st}^{{}} A_{\phi } - 2A_{y\phi } } \right\}} \right]$$, $$E_{5} = \frac{1}{RP}\left[ {1 + \frac{{\theta \left( {\theta + 1} \right)}}{2}\upsilon_{st}^{2} A_{\phi } } \right]$$.

The $MSE\left( {t_{8} } \right)$ at (41) is minimized for

42 $$\left. \begin{gathered} w_{1} = \frac{{\left( {E_{2} E_{4} - E_{3} E_{5} } \right)}}{{\left( {E_{1} E_{2} - E_{3}^{2} } \right)}} \hfill \\ w_{2} = \frac{{\left( {E_{1} E_{5} - E_{3} E_{4} } \right)}}{{\left( {E_{1} E_{2} - E_{3}^{2} } \right)}} \hfill \\ \end{gathered} \right\}\,.$$

Substitution of (42) in (41) provides the minimum *MSE* of $t_{8}$ is given by

43 $$MSE_{\min } \left( {t_{8} } \right) = \overline{Y}^{2} \left[ {1 - \frac{{\left( {E_{2} E_{4}^{2} - 2E_{3} E_{4} E_{5} + E_{1} E_{5}^{2} } \right)}}{{\left( {E_{1} E_{2} - E_{3}^{2} } \right)}}} \right]$$

Now we have the following theorem.

### **Theorem 3.1**

*The* *MSE* *of* $t_{8}$ *is greater than or equal to the minimum* *MSE* *of* $t_{8}$.

$$MSE\left( {t_{8} } \right) \ge MSE_{\min } \left( {t_{8} } \right)$$

## Efficiency comparison

From (2), (4), (6), (8), (16) and (17) we have

44 $$MSE\left( {t_{0} = \overline{y}_{st} } \right) - MSE\left( {t_{3} } \right) = \overline{Y}^{2} A_{y} \rho_{y\phi }^{2} \ge 0,$$

45 $$MSE\left( {t_{1} } \right) - MSE\left( {t_{3} } \right) = \overline{Y}^{2} A_{\phi } \left( {1 - C} \right)^{2} \ge 0,$$

46 $$MSE\left( {t_{2} } \right) - MSE\left( {t_{3} } \right) = \overline{Y}^{2} A_{\phi } \left( {1 + C} \right)^{2} \ge 0,$$

47 $$MSE\left( {t_{1e} } \right) - MSE\left( {t_{3} } \right) = \overline{Y}^{2} \frac{{A_{\phi } }}{4}\left( {1 - 2C} \right)^{2} \ge 0,$$

48 $$MSE\left( {t_{2e} } \right) - MSE\left( {t_{3} } \right) = \overline{Y}^{2} \frac{{A_{\phi } }}{4}\left( {1 + 2C} \right)^{2} \ge 0,$$

It follows from (44) to (46) that the regression estimator *t*_3_ is more efficient than $\overline{y}_{st} ,t_{1,} t_{2} ,t_{1e} \,{\text{and}}\,t_{2e}$.

From (8), (12), (22), (26), (29), (33), (38) and (43) we have

49 $$MSE\left( {t_{3} } \right) - MSE_{\min } \left( {t_{4} } \right) = \overline{Y}^{2} \left[ {A_{y} \left( {1 - \rho_{y\phi }^{2} } \right) + \frac{{\left( {B_{2} B_{4}^{2} - 2B_{3} B_{4} B_{5} + B_{1} B_{5}^{2} } \right)}}{{\left( {B_{1} B_{2} - B_{3}^{2} } \right)}} - 1} \right] \ge 0$$

50 $$MSE\left( {t_{3} } \right) - MSE_{\min } \left( {t_{5} } \right) = \overline{Y}^{2} \left[ {A_{y} \left( {1 - \rho_{y\phi }^{2} } \right) + \frac{{\left( {A_{2} A_{4}^{2} - 2A_{3} A_{4} A_{5} + A_{1} A_{5}^{2} } \right)}}{{\left( {A_{1} A_{2} - A_{3}^{2} } \right)}} - 1} \right] \ge 0$$

51 $$MSE\left( {t_{3} } \right) - MSE_{\min } \left( {t_{6} } \right) = \overline{Y}^{2} \left[ {A_{y} \left( {1 - \rho_{y\phi }^{2} } \right) + \frac{{\left( {A_{2\left( 1 \right)} A_{4\left( 1 \right)}^{2} - 2A_{3\left( 1 \right)} A_{4\left( 1 \right)} A_{5\left( 1 \right)} + A_{1\left( 1 \right)} A_{5\left( 1 \right)}^{2} } \right)}}{{\left( {A_{1\left( 1 \right)} A_{2\left( 1 \right)} - A_{3\left( 1 \right)}^{2} } \right)}} - 1} \right] \ge 0$$

52 $$MSE\left( {t_{3} } \right) - MSE_{\min } \left( {t_{7} } \right) = \overline{Y}^{2} \left[ {A_{y} \left( {1 - \rho_{y\phi }^{2} } \right) + \frac{{\left( {C_{2} C_{4}^{2} - 2C_{3} C_{4} C_{5} + C_{1} C_{5}^{2} } \right)}}{{\left( {C_{1} C_{2} - C_{3}^{2} } \right)}} - 1} \right] \ge 0$$

53 $$MSE\left( {t_{3} } \right) - MSE_{\min } \left( {t_{7\left( m \right)} } \right) = \overline{Y}^{2} \left[ {A_{y} \left( {1 - \rho_{y\phi }^{2} } \right) + \frac{{\left( {D_{2} D_{4}^{2} - 2D_{3} D_{4} D_{5} + D_{1} D_{5}^{2} } \right)}}{{\left( {D_{1} D_{2} - D_{3}^{2} } \right)}} - 1} \right] \ge 0$$

54 $$MSE\left( {t_{3} } \right) - MSE_{\min } \left( {t_{8} } \right) = \overline{Y}^{2} \left[ {A_{y} \left( {1 - \rho_{y\phi }^{2} } \right) + \frac{{\left( {E_{2} E_{4}^{2} - 2E_{3} E_{4} E_{5} + E_{1} E_{5}^{2} } \right)}}{{\left( {E_{1} E_{2} - E_{3}^{2} } \right)}} - 1} \right] \ge 0$$

It follows from (49) to (54) that the classes of estimators $t_{4,} t_{5} ,t_{6} ,\,t_{7} ,t_{7\left( m \right)} \,{\text{and }}t_{8}$ are more efficient than the regression estimator $\,t_{3}$.

Further from (12), (26), (29), (33), (38) and (43) we have

55 $$MSE_{\min } \left( {t_{8} } \right) < MSE_{\min } \left( {t_{4} } \right)\;{\text{if}}\;M_{2} < M_{1} ,$$

56 $$MSE_{\min } \left( {t_{8} } \right) < MSE_{\min } \left( {t_{5} } \right)\;\;{\text{if}}\;M_{3} < M_{1} ,$$

57 $$MSE_{\min } \left( {t_{8} } \right) < MSE_{\min } \left( {t_{6} } \right)\;{\text{if}}\;M_{4} < M_{1} ,$$

58 $$MSE_{\min } \left( {t_{8} } \right) < MSE_{\min } \left( {t_{7} } \right)\;{\text{if}}\;M_{5} < M_{1} ,$$

59 $$MSE_{\min } \left( {t_{8} } \right) < MSE_{\min } \left( {t_{7\left( m \right)} } \right)\;{\text{if}}\;M_{6} < M_{1} ,$$

where $$M_{1} = \frac{{\left( {E_{2} E_{4}^{2} - 2E_{3} E_{4} E_{5} + E_{1} E_{5}^{2} } \right)}}{{\left( {E_{1} E_{2} - E_{3}^{2} } \right)}}$$, $$\,M_{2} = \frac{{\left( {A_{y} - \upsilon_{st}^{2} A_{\phi } A_{y\phi }^{2} - \upsilon_{st}^{2} A_{\phi }^{2} + \upsilon_{st}^{2} A_{\phi }^{2} A_{y}^{{}} } \right)}}{{\left( {A_{\phi } + A_{y} A_{\phi } - \upsilon_{st}^{2} A_{\phi }^{2} - A_{y\phi }^{2} } \right)}}$$, $$\,M_{3} = \frac{{\left( {A_{2} A_{4}^{2} - 2A_{3} A_{4} A_{5} + A_{1} A_{5}^{2} } \right)}}{{\left( {A_{1} A_{2} - A_{3}^{2} } \right)}}$$, $$\,M_{4} = \frac{{\left( {A_{2\left( 1 \right)} A_{4\left( 1 \right)}^{2} - 2A_{3\left( 1 \right)} A_{4\left( 1 \right)} A_{5\left( 1 \right)} + A_{1\left( 1 \right)} A_{5\left( 1 \right)}^{2} } \right)}}{{\left( {A_{1\left( 1 \right)} A_{2\left( 1 \right)} - A_{3\left( 1 \right)}^{2} } \right)}}$$, $$\,M_{5} = \frac{{\left( {C_{2} C_{4}^{2} - 2C_{3} C_{4} C_{5} + C_{1} C_{5}^{2} } \right)}}{{\left( {C_{1} C_{2} - C_{3}^{2} } \right)}}$$, $$M_{6} = \frac{{\left( {D_{2} D_{4}^{2} - 2D_{3} D_{4} D_{5} + D_{1} D_{5}^{2} } \right)}}{{\left( {D_{1} D_{2} - D_{3}^{2} } \right)}}$$.

Therefore we can say that the proposed class of estimators $t_{8}$ is more efficient than the estimators $t_{4,} t_{5} ,t_{6} ,t_{7}$ and $t_{7\left( m \right)}$ as long as the conditions (55), (56), (57), (58), and (59) respectively are satisfied.

## Numerical illustration

To judge the merits of the suggested class of estimators $t_{8}$ over other existing estimators, we have computed the percent relative efficiency (*PRE*) of different estimators with respect to usual unbiased estimator ${\overline{\text{y}}}_{{{\text{st}}}}$ by using the following formulae:

60 $$PRE\left( {t_{1} ,\overline{y}_{st} } \right) = \frac{{A_{y} }}{{\left[ {A_{y} + A_{\phi } \left( {1 - 2C} \right)} \right]}} \times 100,$$

61 $$PRE\left( {t_{2} ,\overline{y}_{st} } \right) = \frac{{A_{y} }}{{\left[ {A_{y} + A_{\phi } \left( {1 + 2C} \right)} \right]}} \times 100,$$

62 $$PRE\left( {t_{3} \,{\text{or}}\,t_{\alpha e} ,\overline{y}_{st} } \right) = \frac{1}{{\left[ {1 - \rho_{y\phi }^{2}} \right]}} \times 100,$$

63 $$PRE\left( {t_{4} ,\overline{y}_{st} } \right) = \frac{{A_{y} }}{{\left[ {1 - M_{2} } \right]}} \times 100,$$

64 $$PRE\left( {t_{1e} ,\overline{y}_{st} } \right) = \frac{{A_{y} }}{{\left[ {A_{y} + \frac{{A_{\phi } }}{4}\left( {1 - 4C} \right)} \right]}} \times 100,$$

65 $$PRE\left( {t_{2e} ,\overline{y}_{st} } \right) = \frac{{A_{y} }}{{\left[ {A_{y} + \frac{{A_{\phi } }}{4}\left( {1 + 4C} \right)} \right]}} \times 100,$$

66 $$PRE\left( {t_{5} ,\overline{y}_{st} } \right) = \frac{{A_{y} }}{{\left[ {1 - M_{3} } \right]}} \times 100,$$

67 $$PRE\left( {t_{6} ,\overline{y}_{st} } \right) = \frac{{A_{y} }}{{\left[ {1 - M_{4} } \right]}} \times 100,$$

68 $$PRE\left( {t_{7} ,\overline{y}_{st} } \right) = \frac{{A_{y} }}{{\left[ {1 - M_{5} } \right]}} \times 100,$$

69 $$PRE\left( {t_{7\left( m \right)} ,\overline{y}_{st} } \right) = \frac{{A_{y} }}{{\left[ {1 - M_{6} } \right]}} \times 100,$$

70 $$PRE\left( {t_{8} ,\overline{y}_{st} } \right) = \frac{{A_{y} }}{{\left[ {1 - M_{1} } \right]}} \times 100,$$

To demonstrate the effectiveness of the proposed class of estimators $t_{8}$, we utilise data on the number of teachers as the study variable (*y*), and the number of students classified as more or less than 750 in both primary and secondary schools as the auxiliary attribute $\phi$ for 923 districts across six regions (as 1: Marmara, 2: Agean, 3: Mediterranean, 4: Central Anatolia, 5: Black Sea, 6: East and Southest Anatolia) in Turkey in 2007 (Source: The Turkish Republic Ministry of Education).

The summary statistics of the data are given in Table 1. We applied Neyman^23^ allocation for allocating the samples to various strata^24^. Source: Koyuncu^9^.

**Table 1:** Data statistics.

Values	Stratum (h)					
	1	2	3	4	5	6
$N_{h}$	127	117	103	170	205	201
$n_{h}$	31	21	29	38	22	39
$\overline{Y}_{h}$	703.74	413.00	573.17	424.66	267.03	393.84
$S_{yh}$	883.83	644.92	1033.46	810.58	403.65	711.72
$S_{\phi h}$	0.213	0.159	0.25	0.31	0.28	0.21
$P_{h}$	0.952	0.97	0.93	0.88	0.91	0.95
$S_{\phi yh}$	25.26	9.98	37.45	44.62	21.04	18.66
$\beta_{{2\left( {\phi h} \right)}}$	16.92	35.57	10.34	4.23	6.67	15.56

It is observed from Table 2 that the estimators ${t}_{1}\text{ and }{t}_{1e}$ are more efficient than the usual unbiased estimator $\overline{y}$ (which does not utilize the auxiliary attribute). The product estimator ${t}_{2}$ and product-type exponential estimator ${t}_{2\left(e\right)}$ perform poor than $\overline{y}$ (due to positive correlation between y and φ). The ${t}_{3}$ is more efficient than ${t}_{1},{t}_{1e},{t}_{2}\text{ and }{t}_{2e}$. The performance of the estimators $\left({t}_{4},{t}_{5},{t}_{6}\right)$ are almost same but marginally better than estimators ${t}_{1},{t}_{1e},{t}_{2},{t}_{2e } \; \text{and } \; {t}_{3}$.

**Table 2:** *PRE*s values of different estimators of $\overline{Y}$ with respect to $\overline{y}$.

Different values of scalars		Estimators										
$a_{st}$	$b_{st}$	$t_{1}$	$t_{1\left( e \right)}$	$t_{2}$	$t_{2\left( e \right)}$	$t_{3}$	$t_{4}$	$t_{5}$	$t_{6}$	$t_{7}$(γ = -1)	$t_{7\left( m \right)}$(γ = -1)	$t_{8}$(δ = -1, η = 1)
$C_{{\phi \left( {st} \right)}}$	NP	101.82	101.75	92.28	96.75	102.00	103.17	103.17	103.17	3366.42	1.71E+10	1.49E+11
1	NP	101.82	101.75	92.28	96.75	102.00	103.17	103.17	103.17	3363.75	1.21E+09	1.06E+10
$\beta_{{2\phi \left( {st} \right)}}$	NP	101.82	101.75	92.28	96.75	102.00	103.17	103.17	103.17	3318.90	6.68E+06	5.84E+07
$C_{{\phi \left( {st} \right)}}$	$\beta_{{2\phi \left( {st} \right)}}$	101.82	101.75	92.28	96.75	102.00	103.17	103.17	103.17	3308.06	4.44E+06	3.88E+07
1	$\beta_{{2\phi \left( {st} \right)}}$	101.82	101.75	92.28	96.75	102.00	103.17	103.17	103.17	3162.20	3.43E+05	3.02E+06
$C_{{\phi \left( {st} \right)}}$	1	101.82	101.75	92.28	96.75	102.00	103.17	103.17	103.17	2786.33	3.47E+04	3.10E+05
1	$C_{{\phi \left( {st} \right)}}$	101.82	101.75	92.28	96.75	102.00	103.17	103.18	103.17	1747.40	2.33E+03	2.02E+04
$\beta_{{2\phi \left( {st} \right)}}$	1	101.82	101.75	92.28	96.75	102.00	103.17	103.18	103.17	1573.86	1.70E+03	1.43E+04
$\beta_{{2\phi \left( {st} \right)}}$	$C_{{\phi \left( {st} \right)}}$	101.82	101.75	92.28	96.75	102.00	103.17	103.18	103.17	1519.10	1.55E+03	1.29E+04
NP	$\beta_{{2\phi \left( {st} \right)}}$	101.82	101.75	92.28	96.75	102.00	103.17	103.18	103.17	1515.26	1.54E+03	1.28E+04
NP	1	101.82	101.75	92.28	96.75	102.00	103.17	103.18	103.17	1499.67	1.50E+03	1.24E+04
NP	$C_{{\phi \left( {st} \right)}}$	101.82	101.75	92.28	96.75	102.00	103.17	103.18	103.17	1498.76	1.50E+03	1.24E+04
1	0	101.82	101.75	92.28	96.75	102.00	103.17	103.18	103.17	1498.43	1.50E+03	1.24E+04

Table 2 also demonstrates that the proposed estimator $t_{8}$ with $\left(\delta =-1,\eta =1,{a}_{st}={C}_{\phi \left(st\right)},{b}_{st}=NP\right)$ has the largest *PRE*(= 1.49E+11) followed by ${t}_{7\left(m\right)}$ with $\left(\lambda =-1,{a}_{st}={C}_{\phi \left(st\right)},{b}_{st}=NP\right)$. It is further observed that the proposed classes of estimators $t_{7\left( m \right)}$ and $t_{8}$ are always better than the classes of estimators $t_{1}$^4^, ${t}_{1e},{t}_{2}\text{ and }{t}_{2e}$, ${t}_{3}$ (difference estimator), $t_{4}$^9^, ${t}_{5}$^3^, ${t}_{6}$^11^, ${t}_{7}$^10^ for all choices of $\left({a}_{st},{b}_{st}\right)$. The proposed class of estimators $t_{8}$ is the best among the estimators closed in Table 2.

Thus, our recommendation is to use the suggested class of estimators ${t}_{7\left(m\right)}$ and ${t}_{8}$ in practice.

## Conclusion

In this article, we propose two classes of estimators for estimating the population mean $\overline{Y}$ of the study variable *y* using information on an auxiliary attribute $\left( \phi \right)$. The suggested classes of estimators are wide-ranging. The biases and mean squared errors of the proposed classes of estimators $t_{7\left( m \right)}$ and $t_{8}$ are derived up to the first degree of approximation. The optimum estimators in the classes of estimators $t_{7\left( m \right)}$ and $t_{8}$ are investigated using the minimum mean squared error formulae. An empirical study is conducted to evaluate the efficiency of the proposed classes of estimators $t_{7\left( m \right)}$ and $t_{8}$ and the findings are presented in Table 2. The results of Table 2 demonstrate that the suggested classes of estimators $t_{7\left( m \right)}$ and $t_{8}$ are more efficient than the recently developed classes of estimators $t_{4} ,t_{5} {,}t_{6} ,t_{7} \;{\text{and}}\;t_{3} \,$ by Koyuncu^9^, Sharma and Singh^3^, Shahzad et al.^11^, Koyuncu^10^, and the difference estimator, with a considerable gain in efficiency. Therefore, we conclude that the proposed classes of estimators and are justified and can be used in practice.

One potential direction for future research is the application of advanced statistical techniques, such as machine learning and artificial intelligence, can be explored to improve the accuracy and efficiency of the estimators. These techniques can also help in identifying relevant auxiliary variables for improving the estimation process. The impact of various sampling designs on the estimation process can be investigated. For example, the effect of unequal sample sizes in different strata, non-response rates, and measurement errors on the accuracy and efficiency of the estimators can be studied. Finally, the extension of the current research to other types of population parameters, such as variance and quantiles, can also be explored. This can lead to the development of new classes of estimators and further improve the accuracy and efficiency of the estimation process.

## Data Availability

All the necessary data generated and/or analysed during the current study are included in this published article.
